# Identification of methotrexate as a heterochromatin-promoting drug

**DOI:** 10.1038/s41598-019-48137-w

**Published:** 2019-08-12

**Authors:** Andre C. Loyola, Lin Zhang, Robin Shang, Pranabananda Dutta, Jinghong Li, Willis X. Li

**Affiliations:** Department of Medicine, University of California San Diego, La Jolla, CA 92093 USA

**Keywords:** Phenotypic screening, Drug development

## Abstract

Heterochromatin is a tightly packed form of DNA involved in gene silencing, chromosome segregation, and protection of genome stability. Heterochromatin is becoming more recognized in tumor suppression and may thus serve as a potential target for cancer therapy. However, to date there are no drugs that are well established to specifically promote heterochromatin formation. Here, we describe a screening method using *Drosophila* to identify small molecule compounds that promote heterochromatin formation, with the purpose of developing epigenetic cancer therapeutics. We took advantage of a *Drosophila* strain with a variegated eye color phenotype that is sensitive to heterochromatin levels, and screened a library of 97 FDA approved oncology drugs. This screen identified methotrexate as the most potent small molecule drug, among the 97 oncology drugs screened, in promoting heterochromatin formation. Interestingly, methotrexate has been identified as a JAK/STAT inhibitor in a functional screen, causing reduced phosphorylation of STAT proteins. These findings are in line with our previous observation that unphosphorylated STAT (uSTAT) promotes heterochromatin formation in both *Drosophila* and human cells and suppresses tumor growth in mouse xenografts. Thus, *Drosophila* with variegated eye color phenotypes could be an effective tool for screening heterochromatin-promoting compounds that could be candidates as cancer therapeutics.

## Introduction

In eukaryotic cells, DNA is folded with histone and non-histone proteins in order to form chromatin. Nucleosomes, the basic units of chromatin consist of 146 bp of DNA wrapped around a histone octomer core and linker histone protein H1, the core being made up of two copies of H2A, H2B, H3 and H4 histone proteins. This chromosomal template is the foundation for many important cellular processes, such as gene transcription, DNA repair and recombination^1,2^. Generally, chromatin is found in two different assortments, euchromatin and heterochromatin. In 1928, Heitz first distinguished between these different chromosomal states cytologically by observing the differential staining of chromatin at interphase. Euchromatin stains lightly at interphase, representing a less condensed, accessible and transcriptionally active form of chromatin, while heterochromatin stains more strongly and is believed to be a more condensed, inaccessible and transcriptionally silent chromatin^1,2^.

Heterochromatin, a condensed and transcriptionally silent form of chromatin, can be further divided into two categories: constitutive and facultative heterochromatin. Constitutive heterochromatin domains occur in regions that are highly dense in repetitive DNA elements and possess structural importance. These regions remain condensed throughout the cell cycle. They are typically found at centromeres and telomeres and help to facilitate separation of chromosomes during mitosis and protect the ends of chromosomes from deterioration, respectively. Facultative heterochromatin domains are important for development and the chromatin state of these regions can change in response to cellular signals and gene activity^1,2^. An example of facultative heterochromatin is X-chromosome inactivation in female mammals, a phenomenon in which one of the X chromosomes is condensed as facultative heterochromatin and formed into what is known as a Barr body^3^.

Various processes, including methylation of CpG islands and post-translational modification of histone proteins, are associated with the dynamic transitions of chromatin states^1,2^. A “histone code” has been described in which particular posttranslational modifications can dictate the chromosomal state^4^. Tri-methylation of histone 3 lysine 9 (H3K9me3) and recruitment of HP1 chromo domain proteins is well established to be associated with heterochromatin and epigenetic silencing^1,2^. These proteins are indicators and markers for heterochromatin formation.

It has become increasingly evident that heterochromatin is relevant to cancer development. It has been shown that in certain biological contexts, HP1 reduction or loss can lead to progression of various types of tumors^5,6^, which could conceivably be due to changes in genomic stability and derepression of genes that normally should be silent. Decreased HP1 levels, which lead to loss of heterochromatin, are also evident in cancer cell lines^5^. In addition, it has been shown that BRCA1, a tumor suppressor gene well established to be associated with breast and ovarian cancer, prevents tumor formation through heterochromatin mediated silencing^7^. Retinoblastoma (Rb) tumor surpressor, a protein that suppresses transcription of E2F genes upon DNA damage in order to prevent the division of damaged cells, also mediates its effects through stabilizing heterochromatin^8–12^.

We have previously identified a non-canonical JAK-STAT pathway in which un-phosphorylated STAT promotes and stabilizes heterochromatin by binding to the HP1 chromodomain protein^13–15^. We have further shown that increasing global heterochromatin levels through expressing HP1a or unphosphorylatable STAT5A can suppress colon cancer growth using a mouse xenograph model. Interestingly, many genes down-regulated by STAT5A and HP1a in common have previously been shown to be overexpressed in or be drivers of human cancers, although genes involved in proliferation and cell death are not significantly affected^16^. These results support the idea that heterochromatin may offer a unique target for human cancers and could offer a treatment that is associated with less side effects compared with the cytotoxic oncology drugs.

For the purpose of identifying compounds that promote heterochromatin formation and for further research as candidates for cancer therapy, we designed and performed a simple screen for small-molecule compounds that can promote heterochromatin formation in *Drosophila*, which is an inexpensive *in vivo* system for drug discovery. These compounds will serve as lead molecules for developing epigenetic cancer drugs in the future.

## Results

### Screening of Oncology Set III using *DX1* flies for heterochromatin-promoting drugs

**Figure 1:**
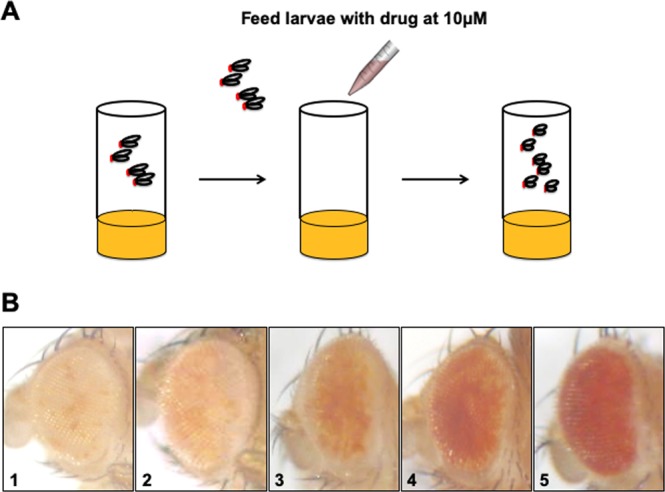
Drug screen using DX1 *Drosophila*. (**A**) Diagram depicting using *Drosophila* to screen for heterochromatin-promoting small molecule compounds. Three *w*^1118^*/Y; DX1/CyO* males and three virgin *w*^1118^ were crossed at room temperature and allowed to lay eggs for two days. Parent flies were then removed and 60 µl of 33% DMSO in water with 10 µM compound was pipetted onto food two days and four days after crosses were first set up. The eye phenotype of *w*^1118^*/Y; DX1*/+ F1 male progeny were then scored. (**B**) Eye phenotype scoring scale. Representative pictures representing the score assigned to flies based on eye color variegation as the following. 1: mostly white with light spots of red/orange scattered in <10% total surface area. 2: Patches of red spots in >10% but <30% of the surface area. 3: Red spots in 50% of surface area. 4: Red spots range from 50% to 70% of eye surface. 5: Red spots occupy >70% of the eye surface.

To identify heterochromatin-promoting drugs, we sought to develop a screening method using *Drosophila*, an inexpensive *in vivo* system (Fig. 1). To this end, we obtained from the National Cancer Institute (NCI) Developmental Therapeutics Program (DTP) a small-molecule drug library, Oncology Set III, consisting of 97 FDA approved oncology drugs (Table S1). We screened this library using the *DX1* strain of *Drosophila melanogaster*. *DX1* flies contain seven tandem copies of a P[mini-*white*^+^] reporter transgene inserted into a euchromatic region of the 2^nd^ chromosome, which induce heterochromatin formation at the insertion locus due to the repetitive nature of the transgene copies, resulting in variegated expression of the P[mini-*white*^+^] reporter transgenes in *white*^−/−^ (*w*^−/−^) genetic background^17,18^. The *white*^+^ gene encodes the transporter that carries precursors of the red and brown pigments into developing eyes during pupation, resulting in red colored eyes when adult flies emerge; flies lacking the *white*^+^ gene have white eyes^19^. Thus the amount of red pigmentation in *DX1* flies is proportional to the level of the *white*^+^ gene products from the P[mini-*white*^+^] transgene. Due to the metastable nature of heterochromatin induced at the P[mini-*white*^+^] tandem repeats, transcription of P[mini-*white*^+^] and thus the degree of variegation in the eye color of *DX1* flies is sensitive to heterochromatin levels. Increasing heterochromatin levels leads to more variegation, less mini-*white*^+^ expression and overall red eye pigmentation; decreasing heterochromatin levels has the opposite effects, resulting in increased red eye pigmentation^14,15,17,18^. Thus, the levels of red pigmentation in *DX1* flies can serve as a convenient readout of heterochromatin levels *in vivo*.

We screened the Oncology Set III library based on the assumption that compounds that promote heterochromatin should cause more variegation in the eye color in *DX1* flies and thus reduced red eye pigmentation, as has been previously reported^14,15,17,18^. For screening, we crossed *DX1/CyO* male flies to *w*^1118^, a commonly used *w*^−/−^ strain in an otherwise wild-type genetic background. The F1 generation was raised in the presence of individual drugs, and the F1 *w*^1118^*; DX1*/+ adult male flies emerged were scored for their red eye color (Fig. 1; see Methods).

### Identification of methotrexate as a heterochromatin-promoting drug

**Figure 2:**
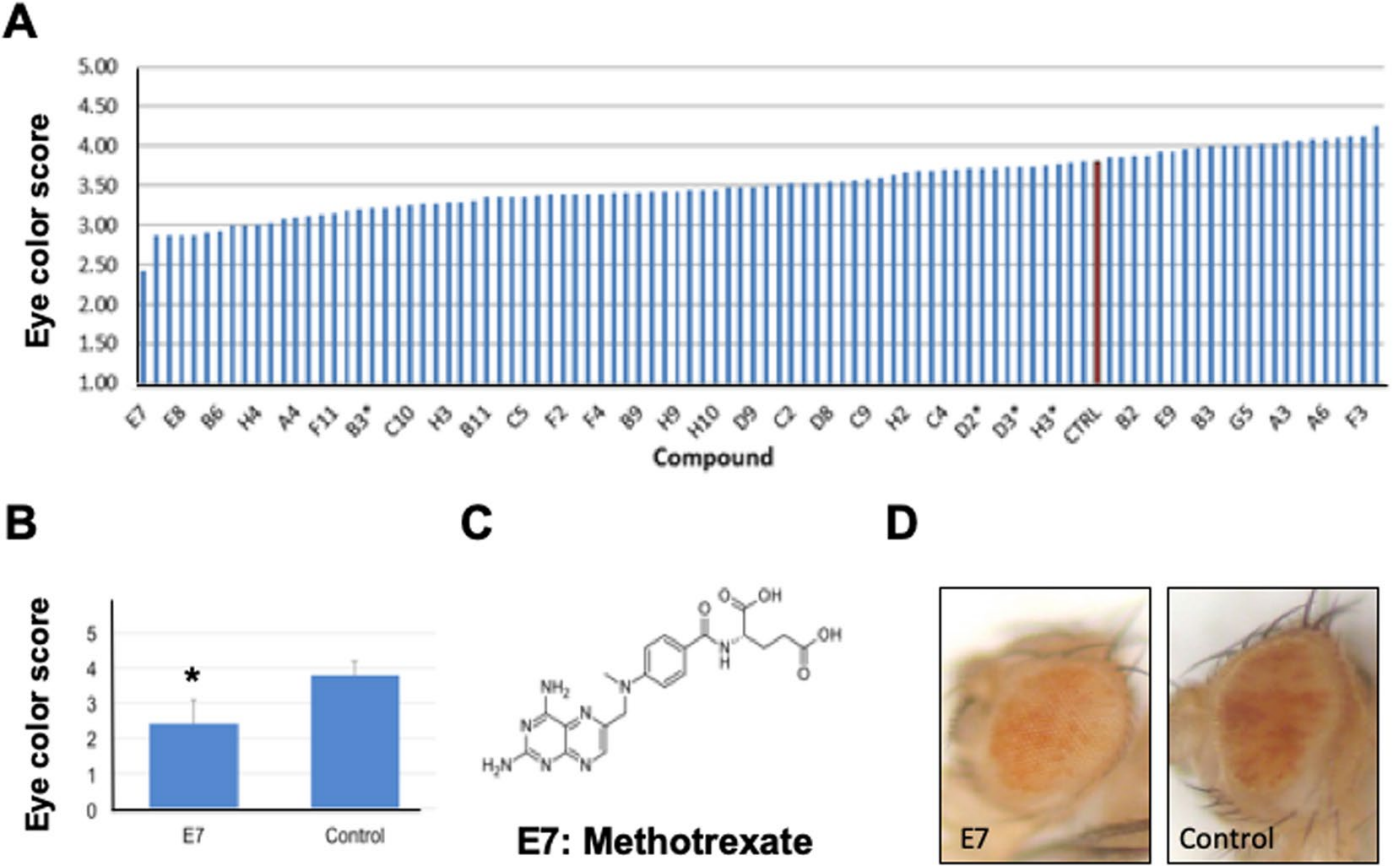
Identification of methotrexate as a heterochromatin-promoting drug. (**A**) Screen of Oncology Set III. Triplicates of each sample were performed represented on bar graph. Control is represented as a red bar. (**B**) Bar graph of E7 and Control. Average score of E7 was 2.43 and average score of control was 3.80. (**C**) Structure of methotrexate, coded as E7 in the library plate (see Table S1). (**D**) Representative pictures of the eyes of E7 (methotrexate) and control *DX1* flies.

We scored each F1 *w*^1118^*/Y; DX1/*+ adult male fly on a scale from 1 to 5, 1 being most variegated and 5 being least variegated (Fig. 1). We then averaged the scores of all the F1 *w*^1118^*/Y*; *DX1/*+ males in each vial. Three trials were done per compound and were represented in a bar graph (Fig. 2A). We identified hit drugs as compounds that received a score that was two standard deviations below the mean of the control, which were exposed to solvent only (33% DMSO in H_2_O). The control flies received a mean score of 3.82 with a standard deviation of 0.67. Therefore a drug that receives a score different from the control score by more than two standard deviations would be considered a hit. The drug that caused the most variegation in this set was methotrexate (4-aminopteroylglutamic acid), with a code name E7 in Plate 1 of the drug library, which received a score of 2.43, less than 2.48 or two standard deviations from the control mean of 3.82 (Fig. 2). Methotrexate was the only drug that received a score two standard deviations below that of the control among all the 97 Oncology Set III drugs screened. We thus pursued methotrexate further as a heterochromatin-promoting drug.

### Methotrexate promotes heterochromatin formation and reduces overproliferation caused by JAK overactivation

**Figure 3:**
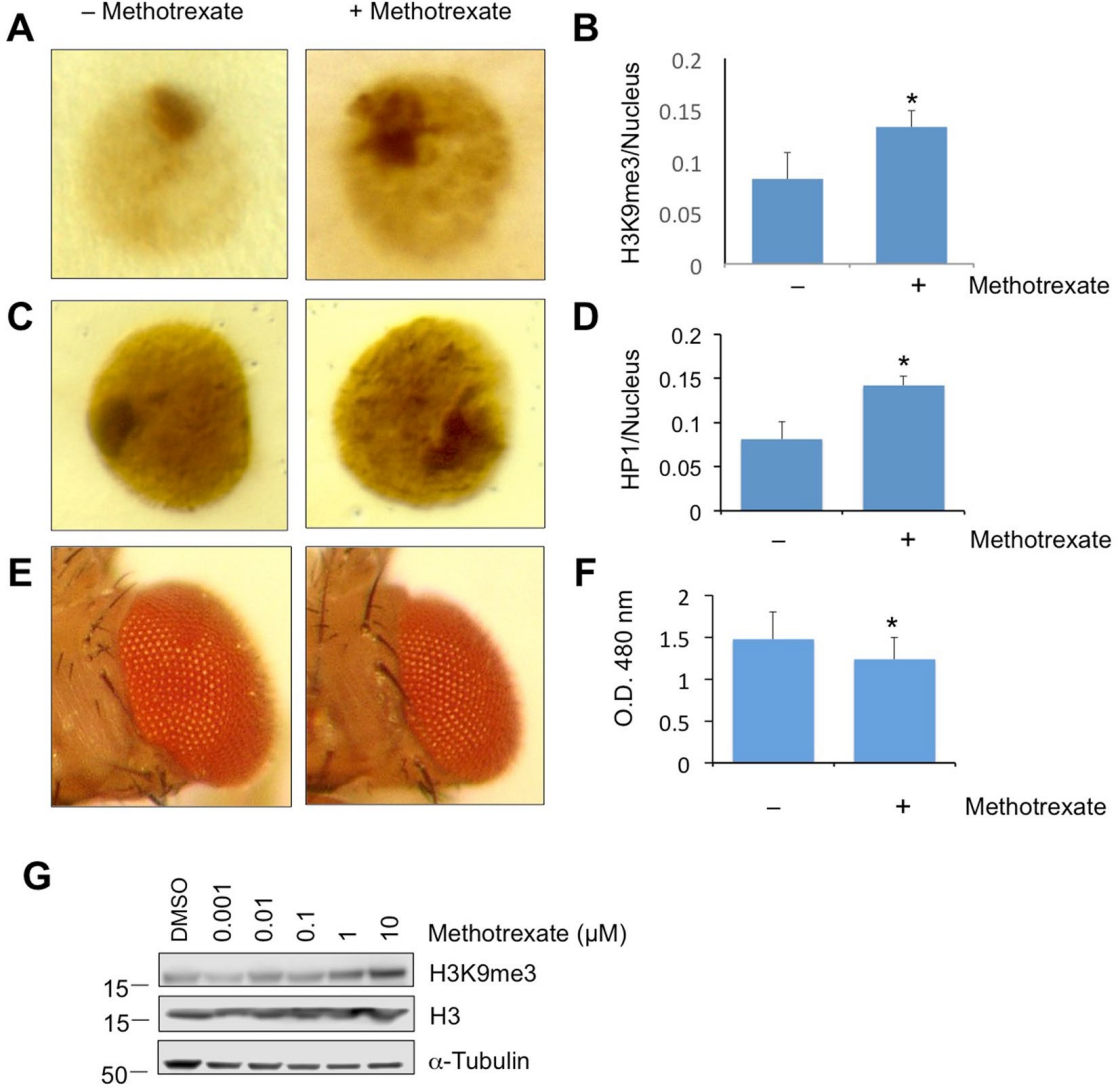
Effects of methotrexate on heterochromatin and cell proliferation. (**A**,**C**,**G**) Flies were raised on food with or without methotrexate at the indicated concentrations (10 µM) in 33%DMSO. Salivary glands from 3^rd^ instar larvae were immunostained with anti-H3K9me3 (**A**) or anti-HP1 (**C**) and photographed with a compound microscope. Heterochromatin levels were quantified as the ratio of H3K9me3-positive area to the area of the nucleus. Representative images are shown. (**G**) Total protein was extracted from 3^rd^ instar larvae without or with methotrexate treatment at the indicated concentrations, and was subjected to SDS-PAGE, following by blotting with antibodies specific for H3K9me3, H3, or α-Tubulin. (**B**,**D**) Quantification of H3K9me3 (**B**) or HP1 (**D**) immunostaining as the ratio of antibody-positive area (dark brown) to the area of the nucleus (light brown). Error bars are standard deviations. *Indicates p < 0.05 by Student’s t-Test. (**E**) Flies carrying the GMR-Gal4 and UAS-Upd transgenes were raised at 25 °C on food with or without 10 µM methotrexate. Adult male flies were photographed to show the eyes. (**F**) Eye volumes were quantified by measuring at O.D. 480 nm the amount of red pigments in fly heads.

To confirm that methotrexate indeed promotes heterochromatin formation, we examined the levels of heterochromatin mark, histone H3 trimethylated at lysine 9 (H3K9me3), after treating larvae with methotrexate. Using immunostaining with anti-H3K9me3 antibodies, we found that H3K9me3 levels were higher in 3^rd^ instar larval salivary gland cells of methotrexate-treated larvae when compared with control larvae treated with solvent only (Figs 3A,B and S1A). *Drosophila* larval salivary glands contain large cells with prominent heterochromatin foci, which allow easy visualization and heterochromatin levels and morphology, as we have previously reported^14,15^. Heterochromatin levels can also be revealed by HP1 immunostaining, as we have previously shown^14^. Indeed, larval salivary gland nuclei show significantly larger HP1-positive heterochromatin area after methotrexate treatment than control (Figs 3C,D and S1B). The effects of methotrexate treatment on H3K9me3 levels are similar to those resulting from genetically altering JAK/STAT signaling, as we have previously shown^14,15^. Lastly, using Western blotting, we confirmed that methotrexate treatment causes an increase in levels of H3K9me3 in treated larvae (Fig. 3G). Thus, we are able to confirm at cellular and protein level, using immunostaining, that methotrexate promotes heterochromatin formation.

Methotrexate is one of the first chemotherapy agents used for cancer treatment, and it is also used as an immunosuppresant to treat rheumatoid arthritis^20,21^. Methotrexate is believed to be an antimetabolite that acts as a competitive inhibitor of dihydrofolate reductase (DHFR)^22^. Interestingly, in a screen for small molecules that modulate JAK/STAT signaling using cells, methotrexate was identified as a JAK/STAT pathway inhibitor, which reduces both STAT reporter gene expression in *Drosophila* cells and STAT5 phosphorylation in human cells^23^.

To test the effects of methotrexate on JAK/STAT signaling in flies, we used transgenic flies that overexpress the JAK/STAT ligand Unpaired (Upd) in the developing eye (GMR > Upd), resulting in larger eyes when adult flies emerge (Fig. 3E)^14,24^. We found that, treating the larvae with methotrexate at the same concentration that causes increased heterochromatin, reduced the eye size of GMR > Upd flies (Fig. 3F). This result is consistent with the idea that methotrexate reduces JAK/STAT signaling. However, we cannot rule out the possibility that the observed reduction in GMR > Upd fly eyes was due to decreased cell proliferation caused by inhibition of DHFR by methotrexate, as previously known^22^. Nonetheless, taken together with our previous finding that uSTAT promotes heterochromatin formation^13,15^, it is possible that methotrexate reduces pSTAT levels and thus increases relative uSTAT levels, resulting in higher levels of heterochromatin.

## Discussion

Heterochromatin formation represents a potential epigenetic tumor suppression mechanism^2,5,7,16^. Compounds that promote heterochromatin formation are therefore desirable as candidate cancer therapeutic agents. In order to identify new heterochromatin-promoting small molecule drugs, we performed a simple screen using *Drosophila*, taking advantage of the *DX1* strain that exhibits a heterochromatin-dependent eye color variegation phenotype (Fig. 1). Upon screening a small library of 97 drugs, we identified a small molecule drug, methotrexate, as causing the highest increase in variegation, which is significantly different from control (Fig. 2).

Interestingly, as a first-generation cancer chemotherapy drug and known to be a dihydrofolate reductase (DHFR) inhibitor, methotrexate has more recently been identified as a specific JAK/STAT pathway inhibitor, resulting in a reduced level of pSTAT without changing total STAT levels, therefore causing a relative increase in uSTAT levels^23^. We have previously shown that reducing pSTAT and increasing uSTAT levels promote heterochromatin formation as a non-canonical function of STAT^13,15,16^. Thus, the identification of methotrexate as a JAK/STAT inhibitor^23^ and as a heterochromatin-promoting drug (this study) is line with our research demonstrating that uSTAT promotes heterochromatin formation^15,16^.

Our screening method is relatively straightforward and easy to carry out. However, there is a caveat when calculating the drug concentration used in the screening. We added drug solutions at 10 µM to the top of the food media, which is in semisolid state. The effective drug concentration is expected to remain in a steep gradient, with the top portion close to 10 µM and limited penetration to the bottom of the food. On the other hand, larvae mostly stay on and consume the top portion of the food. Thomas *et al*. have shown that methotrexate inhibits STAT phosphorylation in cultured cells at concentrations ranging from 0.01 to 50 µM with minimal toxicity^23^. Thus, we assume that our drugging regimen for methotrexate effectively inhibited STAT phosphorylation without strong toxic effects. Indeed, at 10 µM concentration, we did not observe much lethality in larvae treated with any of the 97 drugs we used in screening. However, when added at 100 µM concentration, methotrexate did cause lethality to the larvae. Thus, 10 µM is a tolerable concentration for screening these small-molecule drugs.

We will expand the screening effort for heterochromatin-promoting substances to a larger pool of small molecule compounds using the *Drosophila* variegated eye phenotype. Upon discovery of compounds that are suspected to promote heterochromatin formation, we will identify the mechanisms by which these drugs accomplish this in future research. We will also investigate the ability and the underlying mechanisms by which these compounds to suppress cancer.

## Methods

### Small-molecule drug library

Compound Libraries were obtained through the National Cancer Institute (NCI) Developmental Therapeutics Program. We screened Oncology Set III, a set of 97 oncology drugs that have been previously approved by the US Food and Drug Administration (FDA). The compounds were delivered in 96-well polypropylene (PP) microtiter plates with 60 compounds per plate. The plates were stored dry at −20 °C and contained 0.20 µMol of compound + 1 µL of glycerol. 19 µL of DMSO was added to each well to obtain 20 µl of 10 mM stock solutions.

### *Drosophila* screening method

*DX1* flies were kindly provided by James Birchler (University of Missouri)^17,25^. Three *w*^1118^*/Y; DX1/CyO* male flies and three virgin *w*^1118^ female flies were crossed in a 25 × 95 mm food vial with 9 ml of standard food (Bloomington recipe). Flies were allowed to lay eggs for two days at room temperature. Parent flies were then removed from the vial and 60 µl of a 10 µM drug solution was pipetted onto food on day two, and another 60 µl of the same drug solution was added again on day four after crosses were set up (Fig. 1A). All drug compounds were prepared by diluting the original stock (10 mM in DMSO) to a 10 µM final concentration by dissolving in 33% DMSO in water. Since the fly food is in solid state, the drug solution is expected to remain in a deep gradient, with the top portion close to 10 µM and limited penetration to the bottom of the food. For control treatment, only 33% DMSO in water was used.

Scoring was carried out 2 days after the F1 flies emerge, all *w*^1118^*/Y; DX1*/+ heterozygous males, identified by lacking the *CyO* dominant curly wing phenotype, were scored for eye color on a scale of 1 to 5 (see Fig. 1B). (The choice of 1 to 5 scale was based on Sun Tzu’s writing in the Art of War, “The colors do not exceed five, but the changes of the five colors can never be completely seen.”) Color scale is determined by the following rules.

1. Mostly white with light spots of red/orange scattered in <5% total surface area.
2. Patches of red spots in >5% but <25% of the surface area.
3. Red spots in >25% to <50% of surface area.
4. Red spots range from >50% to <75% of eye surface.
5. Red spots occupy >75% of the eye surface.

Triplicates of each cross were performed. Eye color for all F1 males of the *w*^1118^*/Y*; *DX1*/+ genotype were assigned from 1 to 5 as above. Color index was calculated as the average of all the males scored. Representative pictures were taken using a digital camera through microscope lens.

### Eye pigmentation measurement

To measure red eye pigment levels, the heads of 40 male flies (2 days old, raised at 25 °C) of appropriate genotypes were severed and homogenized in methanol (1 ml, acidified with 0.1% HCl). Eye pigmentation was represented as the absorbance of the supernatant at 480 nm.

### Salivary gland immunostaining and western blotting

The salivary gland dissection and whole-mount immunostaining procedure was adopted from published protocols^26^ and as previously described^14^. Briefly, salivary glands were dissected from wandering 3^rd^ instar larvae by grasping and pulling the mouth hook with forceps in *Drosophila* Ringer’s solution. The larvae had been treated with drug solution or control solvent, as described above. The dissected salivary glands were fixed in 4% paraformaldehyde and 0.3% Triton-X in phosphate buffered salient (PBS) for 20 min, were then blocked with 1% bovine serum albumin (BSA) in PBS, and were incubated with primary antibodies at 4 °C for overnight and then with HRP-conjugated second antibodies. Histochemical staining was done following routine procedures. Stained salivary gland tissues were photographed with a Zeiss epifluorescence microscope with Nomarski optics. Images were cropped and minimally processed with Adobe Photoshop.

Mouse monoclonal anti-HP1 (C1A9; 1:50) was from Developmental Hybridoma Bank (University of Iowa). Rabbit antibodies against H3K9me3 (07-442; 1:250) with cross species specificities were from MilliporeSigma (Burlington, MA). HRP conjugated anti-rabbit IgG secondary antibodies were used in whole-mount immunostaining and Western blotting.

## Supplementary information

Supplementary Table S1

Supplementary Figures
