# Supplementary material for: Identification of methotrexate as a heterochromatin-promoting drug
Source: Sci Rep. 2019 Aug 12;9:11673. doi: 10.1038/s41598-019-48137-w (PMC6690983; doi:10.1038/s41598-019-48137-w)
Supplement: Supplementary file 1 — Supplementary Table S1 [file 41598_2019_48137_MOESM1_ESM.pdf]

Supplementary Information

Identification of methotrexate as a heterochromatin-promoting drug

Andre C. Loyola, Lin Zhang, Robin Shang, Pranabananda Dutta, Jinghong Li, & Willis X. Li §

Department of Medicine, University of California San Diego, La Jolla, CA 92093

§ corresponding author (wxli@ucsd.edu)

Running title: Methotrexate promotes heterochromatin formation

Keywords: Methotrexate, heterochromatin, *Drosophila*, JAK/STAT, drug screen, position-effect variegation

Supplementary Table 1. National Cancer Institute Developmental Therapeutics Set III

AODIII_Plate1-4740

Well_ID	No.	NSC	CAS	DRUG NAME	MW
A02	2	1390-R	315-30-0	Allopurinol	136
A03	3	45388-R	4342-03-4	Dacarbazine	182
A04	4	409962-T	154-93-8	Carmustine	214.05
A05	5	109724-X	3778-73-2	Ifosfamide	261.09
A06	6	38721-U	53-19-0	Mitotane	320
A07	7	25154-X	54-91-1	Pipobroman	356.06
A08	8	226080-W	53123-88-9	Rapamycin	914.18
A09	9	713563-U	107868-30-4	Exemestane	296
A10	10	712807-X	154361-50-9	Capecitabine	359
A11	11	747971-U	284461-73-0	Sorafenib	464.82
B02	14	19893-G	51-21-8	Fluorouracil	130
B03	15	92859-Q	1327-53-3	Arsenic trioxide	198
B04	16	26271-N	50-18-0	Cyclophosphamide	261
B05	17	119875-I	15663-27-1	Cisplatin	300.06
B06	18	82151-A	23541-50-6	Daunorubicin HCl	564
B07	19	71423-Q	595-33-5	Megestrol acetate	385
B08	20	3053-Y	50-76-0	Dactinomycin	1255.43
B09	21	719344-F	120511-73-1	Anastrozole	293.37
B10	22	719627-M	169590-42-5	Celecoxib	381
B11	23	747974-X	84449-90-1	Raloxifene HCl	473.59
C02	26	32065-L	127-07-1	Hydroxyurea	76.05
C03	27	362856-R	85622-93-1	Temozolomide	194.15
C04	28	34462-Q	66-75-1	Uracil mustard	252.1
C05	29	122758-Q	302-79-4	Tretinoin	300.44
C06	30	141540-H	33419-42-0	Etoposide	589
C07	31	241240-Z	41575-94-4	Carboplatin	371
C08	32	608210-H	125317-39-7	Vinorelbine tartrate	1079
C09	33	719345-G	112809-51-5	Letrozole	285
C10	34	750690-Z	557795-19-4	Sunitinib	398
C11	35	719276-G	129453-61-8	Fulvestrant	606.75
D02	38	752-W	154-42-7	Thioguanine	167
D03	39	750-U	55-98-1	Busulfan	246.29
D04	40	63878-P	69-74-9	Cytarabine HCl	280
D05	41	169780-A	24584-09-6	Dexrazoxone	268.27
D06	42	180973-S	54965-24-1	Tamoxifen citrate	563.65
D07	43	266046-O	61825-94-3	Oxaliplatin	397
D08	44	102816-P	320-67-2	Azacitidine	244.21
D09	45	721517-Q	118072-93-8	Zolendronic acid	272
D10	46	279836-C	65271-80-9	Mitoxantrone	444
D11	47	24559-A	18378-89-7	Plicamycin	1085.16
E02	50	755-Z	50-44-2	Mercaptopurine	152
E03	51	13875-O	645-05-6	Altretamine	210
E04	52	66847-R	50-35-1	Thalidomide	258.23
E05	53	218321-O	53910-25-1	Pentostatin	268.27

E06	54	702294-V	52205-73-9	Estramustine disodium phos	566
E07	55	740-K	59-05-2	Methotrexate	454.44
E08	56	127716-G	2353-33-5	Decitabine	228.21
E09	57	747972-V	191732-72-6	Lenalidomide	259.26
E10	58	715055-R	184475-35-2	Gefitinib	446.9
E11	59	49842-J	143-67-9	Vinblastine sulfate	909
F02	62	762-J	55-86-7	Nitrogen mustard	192.52
F03	63	27640-Z	50-91-9	Floxuridine	246.19
F04	64	77213-K	366-70-1	Procarbazine	257.76
F05	65	613327-S	122111-03-9	Gemcitabine HCl	299.65
F06	66	698037-T	137281-23-3	Pemetrexed	427.42
F07	67	14229-X	69-05-6	Acrichine	473
F08	68	296961-R	20537-88-6	Amifostine	214.22
F09	69	606869-X	123318-82-1	Clofarabine	304
F10	70	718781-R	183319-69-9	Erlotinib HCl	430
F11	71	369100-F	99011-02-6	Imiquimod	240.31
G02	74	6396-J	52-24-4	Thiotepa	189
G03	75	45923-X	298-81-7	Methoxsalen	216
G04	76	85998-J	18883-66-4	Streptozocin	265.22
G05	77	3088-N	305-03-3	Chlorambucil	304.22
G06	78	122819-I	29767-20-2	Teniposide	656.66
G07	79	609699-Y	119413-54-6	Topotecan HCl	457.91
G08	80	686673-R	121032-29-9	Nelarabine	297
G09	81	312887-C	75607-67-9	Fludarabine	365.21
G10	82	732517-W	863127-77-9	Dasatinib	488
G11	83	9706-H	51-18-3	Triethylenemelamine	204
H02	86	18509-Z	5451-09-2	Aminolevulinic acid	167.59
H03	87	79037-R	13010-47-4	Lomustine, CCNU	233.7
H04	88	105014-F	4291-63-8	Cladribine	286
H05	89	26980-J	50-07-7	Mitomycin C	334.33
H06	90	616348-A	100286-90-6	Irinotecan HCl	623.15
H07	91	628503-O	114977-28-5	Docetaxel	807.89
H08	92	701852-Q	149647-78-9	Vorinostat	264
H09	93	681239-L	179324-69-7	Bortezomib	384.24
H10	94	743414-R	152459-95-5	Imatinib	493.61
H11	95	8806-C	3223-07-2	Melphalan	341.66

AODIII_plate2-4741-1

Well_ID	No.	NSC	CAS	DRUG NAME	MW
A02	2	67574-I	2068-78-2	Vincristine sulfate	923.04
A03	3	745750-H	231277-92-2	Lapatinib	581.06
A04	4	756645-X	877399-52-5	Crizotinib	450.3
B02	14	125066-Y	11056-06-7	Bleomycin	1512.61
B03	15	138783-K	3543-75-7	Bendamustine HCl	394.7
C02	26	125973-L	33069-62-4	Paclitaxel	853.92
C03	27	737754-P	635702-64-6	Pazopanib HCl	473.9
D02	38	246131-R	56124-62-0	Valrubicin	723.64
D03	39	754143-F	128517-07-7	Romidepsin	540.6
E02	50	733504-U	159351-69-6	Everolimus	958

E03	51	754230-X	146464-95-1	Pralatrexate	477.4
F02	62	747599-Q	641571-10-0	Nilotinib	529.51
F03	63	760766-C	443913-73-3	Vandetanib	475.3
G02	74	747973-W	219989-84-1	Ixabepilone	506.7
G03	75	761431-Z	918504-65-1	Vemurafenib	489.9
H02	86	123127-R	25316-40-9	Doxorubicin HCl	579.99
H03	87	761432-A	183133-96-2	Cabazitaxel	835.9

WELL_ID	The grid ID for the well (A02, H11, etc.)
NSC	The compound ID number, which we use to key the compound in the database.
CAS	Chemical Abstract Service identification number
MW	Molecular Weight
