# Supplementary material for: Identification of methotrexate as a heterochromatin-promoting drug
Source: Sci Rep. 2019 Aug 12;9:11673. doi: 10.1038/s41598-019-48137-w (PMC6690983; doi:10.1038/s41598-019-48137-w)
Supplement: Supplementary file 2 — Supplementary Figures [file 41598_2019_48137_MOESM2_ESM.pdf]

Supplementary Figures

Identification of methotrexate as a heterochromatin-promoting drug

Andre C. Loyola, Lin Zhang, Robin Shang, Pranabananda Dutta, Jinghong Li, & Willis X. Li [§]

Department of Medicine, University of California San Diego, La Jolla, CA 92093

§ corresponding author (wxli@ucsd.edu)

Running title: Methotrexate promotes heterochromatin formation

Keywords: Methotrexate, heterochromatin, *Drosophila*, JAK/STAT, drug screen, position-effect variegation

Figure S1

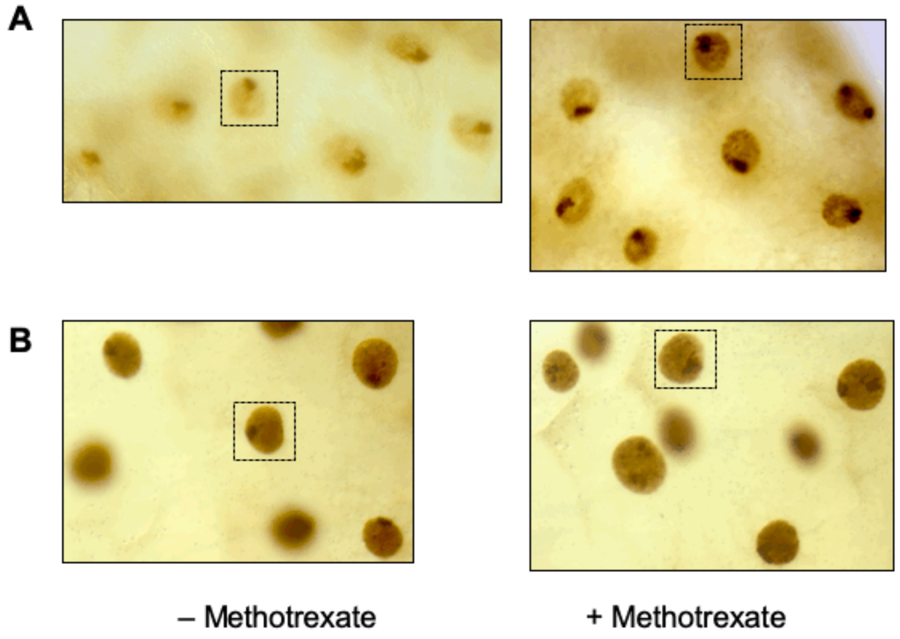

Figure S1. Effects of methotrexate on salivary gland heterochromatin

Flies were raised on food with (lower panel) or without (upper panel) methotrexate at 10μM in 33%DMSO. Salivary glands from 3rd instar larvae were immunostained with anti-H3K9me3 (A) or anti-HP1 (B), and were photographed with a compound microscope. Heterochromatin levels were quantified as the ratio of H3K9me3-positive or HP1-positive area to the area of the nucleus. Representative images with approximately six nuclei of each type are shown.

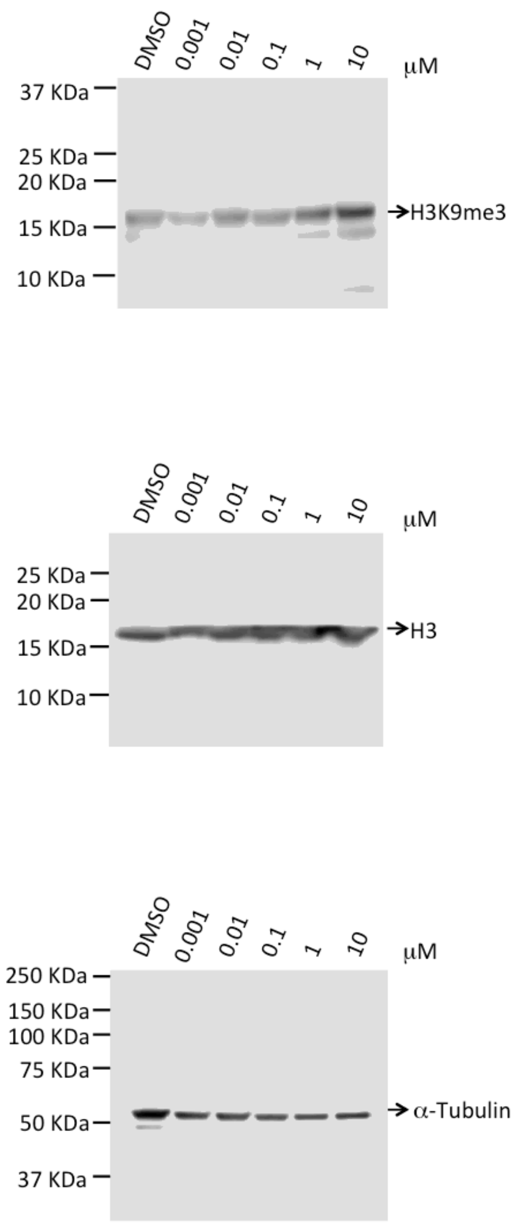

Figure S2. Full-length protein gels

Full-length protein gels for the cropped version shown in Figure 3G.
